# Sweet spot mapping and structural connectivity in subthalamic stimulation: predicting neuropsychiatric outcomes in Parkinson’s disease

**DOI:** 10.3389/fnins.2025.1577588

**Published:** 2025-04-28

**Authors:** Jiuqi Yan, Jian Sun, Xiang Wei, Chang Qiu, Liang Zhao, Bei Luo, Wenwen Dong, Jingxuan Liu, Guanghan Lu, Wenbin Zhang

**Affiliations:** Department of Functional Neurosurgery, The Affiliated Brain Hospital of Nanjing Medical University, Nanjing, China

**Keywords:** Parkinson’s disease, deep brain stimulation, volume of tissue activated, sweet spot mapping, connectomes

## Abstract

**Objective:**

STN-DBS is an effective treatment for Parkinson’s disease (PD), improving motor symptoms, but its impact on non-motor symptoms, such as anxiety and depression, remain unclear. This study investigates the relationship between electrode contact locations, their corresponding volume of tissue activated (VTA), and postoperative changes in emotional symptoms. It aims to identify optimal group-level stimulation sites for improving anxiety and depression in PD patients and to develop a structural connectome to explore how cortical regions targeted by fiber projections correlate with mood outcomes.

**Methods:**

We retrospectively studied 56 PD patients who underwent bilateral STN-DBS, assessed 6 months post-surgery. Standardized scales evaluated motor, affective, and cognitive symptoms before and after the procedure. Electrode positions were reconstructed using Lead-DBS, and VTAs were calculated. Voxel-wise sweet spot and structural connectivity analyses investigated how stimulation sites influenced clinical outcomes.

**Results:**

Compared to preoperative assessments, postoperative evaluations revealed varying degrees of improvement in motor function, quality of life, and symptoms of anxiety and depression in PD patients (*p* < 0.05). The amelioration of anxiety and depression was associated with electrode contacts located in the ventral region of the STN. Specifically, improvements in anxiety were positively correlated with the VTA in the limbic region of the right STN. Sweet spot analysis revealed that stimulation of the ventrocentral region of the left STN was significantly associated with emotional improvement. Structural connectivity analysis revealed that fiber tracts to the prefrontal cortex (PFC) were positively associated with anxiety and depression improvement, while those to the sensorimotor cortex (SMC) showed a negative correlation.

**Conclusion:**

STN-DBS markedly improves motor symptoms and quality of life in PD patients while also positively impacting anxiety and depressive symptoms. The ventral STN is likely the optimal stimulation target for ameliorating anxiety and depressive symptoms. The therapeutic effects of STN-DBS electrodes may promote postoperative improvements in anxiety and depression by modulating fiber tracts connected to prefrontal regions. Future research should leverage connectome mapping and isolated fiber tracts to refine electrode placement, using directional leads to target specific STN subregions for improved symptom management.

## 1 Introduction

Parkinson’s disease (PD) is a chronic, progressive neurodegenerative disorder characterized by a complex interplay of pathological processes, including abnormal aggregation of α-synuclein, mitochondrial dysfunction, and neuroinflammation (Bloem et al., 2021). The cardinal motor symptoms of PD encompass bradykinesia, resting tremor, rigidity, and postural instability. In addition to motor manifestations, PD patients often experience non-motor symptoms such as anxiety and depression, which can significantly exacerbate overall disability and disease burden (Pontone and Mills, 2021).

Deep brain stimulation (DBS) has emerged as an effective surgical treatment for PD, demonstrating improvements in motor function and quality of life, and is increasingly being adopted by PD patients (Meng et al., 2023; Jost et al., 2024). The subthalamic nucleus (STN) is currently the most common and preferred target for DBS in PD (Hariz and Blomstedt, 2022). The STN is a component of the basal ganglia, a dense neural network involved in the regulation of motor, cognitive, motivational, and emotional functions (van Wijk et al., 2020; Kühn et al., 2005). The functional segregation hypothesis of the STN proposes a tripartite model, categorizing the STN into sensorimotor (dorsolateral), cognitive/associative (intermediate), and limbic (ventromedial) subregions. These three subregions are not completely independent but exhibit a degree of overlap and integration (Haynes and Haber, 2013; Lambert et al., 2012). STN DBS has been shown to alleviate motor symptoms in PD patients and even induce changes in emotional and cognitive functions; however, the extent of these improvements varies. Although most studies suggest that STN-DBS improves anxiety and depressive symptoms in PD patients, the risk of emotional deterioration remains (Witt et al., 2013; Kurtis et al., 2017; Bucur and Papagno, 2023). This risk is closely linked to individual differences, electrode placement, and postoperative medication adjustments. Notably, stimulation of different STN subregions may differentially influence the activity of the STN fiber projection areas, resulting in corresponding stimulation effects and varying degrees of impact on patients’ motor and neuropsychological functions (Petry-Schmelzer et al., 2019; Dafsari et al., 2018; Eisenstein et al., 2014). Therefore, selecting appropriate stimulation contacts and optimizing stimulation parameters is crucial.

Previous studies have investigated the effects of STN electrode contact location on both motor and non-motor symptoms in PD patients (Gourisankar et al., 2018; Vitek et al., 2022). Additional studies have integrated the volume of tissue activated (VTA) with electrode contact positioning to explore its effects on motor symptoms, quality of life, and non-motor symptoms such as mood and cognition (Tödt et al., 2022; Liang et al., 2023). With advancements in technology and methodology, an increasing number of researchers have adopted cutting-edge approaches, such as sweet spot mapping and DBS fiber filtering. These techniques enable in-depth data analysis at both local and global network levels, precisely elucidating dysfunctional circuits related to the disease. For instance, studies have utilized these methods to identify optimal stimulation sites and associated connectivity networks for treating freezing of gait in PD (Fan et al., 2023), as well as to investigate the neuroimaging mechanisms underlying the effects of DBS on sleep in PD patients (Xue et al., 2023). However, research utilizing sweet spot mapping and DBS fiber filtering to explore the effects of DBS on anxiety and depression in PD remains limited.

This study integrates DBS with connectomics to address the following primary objectives: (1) to evaluate the effects of bilateral STN-DBS on motor function, quality of life, and mood in PD patients; (2) to investigate the relationship between electrode contact locations, their corresponding VTA, and postoperative mood changes, thereby identifying group-level optimal stimulation sites for alleviating anxiety and depression in PD; and (3) to employ DBS fiber filtering to construct a structural connectome seeded from the optimal stimulation sites, examining the correlations between cortical projections of structural fiber bundles and improvements or worsening of anxiety and depression. By combining theoretical insights with practical applications, this research aims to provide novel perspectives on the neuroimaging mechanisms of DBS in PD treatment and to offer evidence-based guidance for optimizing clinical therapeutic strategies.

## 2 Materials and methods

### 2.1 Patients

This retrospective study analyzed 60 PD patients who underwent bilateral STN DBS at the Affiliated Brain Hospital of Nanjing Medical University between July 2021 and February 2023. All surgeries were performed by the same neurosurgeon to maintain surgical consistency. Of the initial cohort, 2 patients were excluded due to incomplete neuropsychiatric assessments, and 2 were excluded due to insufficient quality of imaging data, which led to errors in electrode reconstruction. After excluding patients with missing imaging data or those lost to follow-up, the final cohort comprised 56 patients. In accordance with the MDS clinical diagnostic criteria (Postuma et al., 2015), all patients were diagnosed with PD by two neurologists. The inclusion criteria for deep brain stimulation (DBS) surgery were as follows: (1) age ≤ 75 years; (2) disease duration ≥ 4 years; (3) Hoehn–Yahr stage ranging from 2.5 to 5.0 during the “off” period of anti-PD medication; (4) significant reduction in medication efficacy or the presence of “on-off” phenomena, motor fluctuations, or dyskinesia, adversely impacting the patients’ quality of life; (5) improvement rate ≥ 30% as assessed by the acute levodopa challenge test. Exclusion criteria included: (1) patients with concomitant organic central nervous system disorders or structural lesions (e.g., epilepsy, brain tumors, or cerebrovascular diseases); (2) subjects unable to cooperate with clinical assessments due to cognitive or behavioral impairments; and (3) patients with incomplete follow-up data. The study was approved by the Ethics Committee of the Affiliated Brain Hospital of Nanjing Medical University, and informed consent was obtained from all patients or their families. All procedures were conducted in accordance with the Declaration of Helsinki.

### 2.2 Surgical procedure and postoperative programming

On the day of surgery, patients were fitted with a stereotactic frame and underwent a cranial CT scan. The CT images were fused with MRI images to select the STN as the target and design the electrode implantation trajectory. We employed intraoperative microelectrode recording technique to identify the entry and exit points of typical STN discharge patterns, ensuring accurate electrode placement. After target confirmation, we implanted a four-contact electrode (model L301, PINS, Peking, China) with the most distal contact positioned at the inferior border of the STN. Following bilateral implantation of the deep brain electrodes, pulse generators were placed in the subclavicular region and connected to the intracranial electrodes. Postoperative CT scans were performed within 24 h after surgery to exclude intracranial hemorrhage and were fused with preoperative MRI images to confirm accurate electrode placement. Typically, the stimulation system was turned on and programmed one month after surgery. Each electrode contact within the bilateral STN was tested in a monopolar mode, and the optimal contact and stimulation parameters were selected based on the patient’s symptomatic changes and adverse reactions. Subsequently, stimulation parameters were adjusted according to the patient’s symptom control, and medication regimens were modified as necessary. All postoperative programming procedures were performed by two experienced movement disorder specialists (C.Q. and W.D.).

### 2.3 Clinical assessment

Motor function was assessed using the MDS Unified Parkinson’s Disease Rating Scale Part III (MDS-UPDRS-III) in patients under preoperative medication-off (MED OFF) conditions and at 6 months postoperatively in both MED OFF and continuous DBS on (STIM ON) states. Quality of daily life was evaluated using the 39-item Parkinson’s Disease Questionnaire (PDQ-39) at the same time points. Anxiety and depressive symptoms were evaluated using the Hamilton Anxiety Scale (HAMA) and Hamilton Depression Scale (HAMD), respectively. Patients’ medication regimens were recorded concurrently and converted to levodopa equivalent daily dose (LEDD) (Jost et al., 2023). MED OFF was defined as the state in which PD patients were off dopamine agonists for 72 h and off levodopa preparations for 12 h. STIM ON referred to the state 30 min after turning on the implantable pulse generator (IPG). Improvement rates for motor function, anxiety, and depressive symptoms were calculated using the MDS-UPDRS-III, HAMA, and HAMD scales, respectively, as [(preoperative score—postoperative score)/preoperative score] × 100%.

### 2.4 Image analysis

**FIGURE 1:**
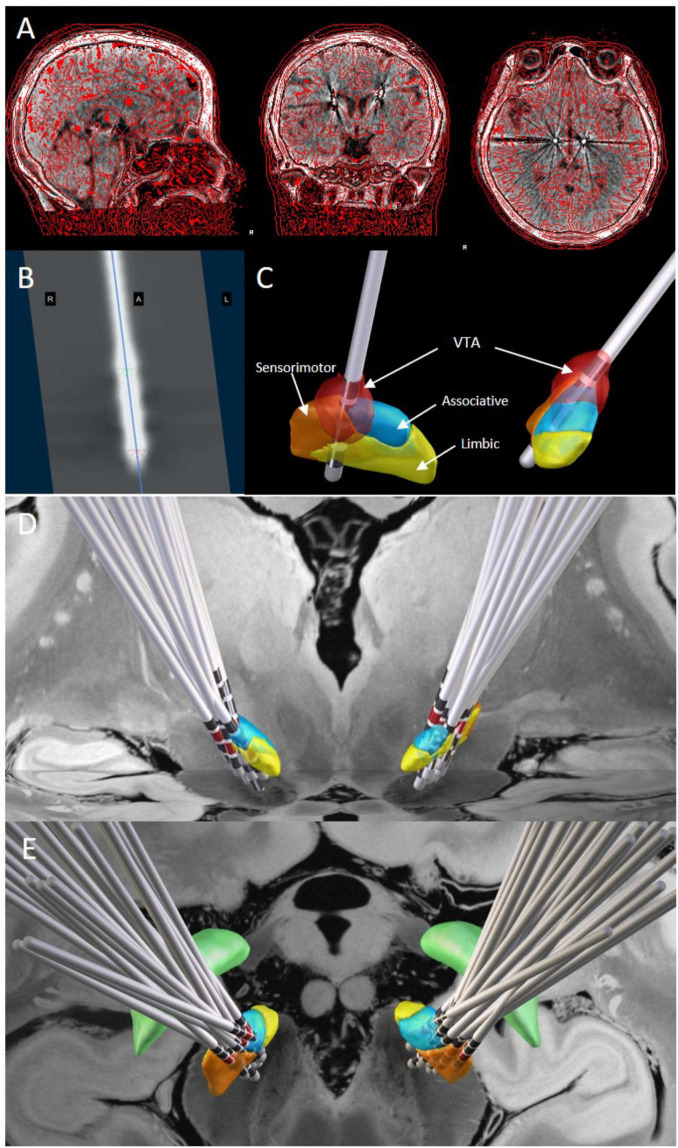
DBS lead localization and volume of tissue activated estimation. **(A)** The fusion result of postoperative thin-slice cranial CT and preoperative MR images. **(B)** The electrode image formed by tracking the electrodes using the PaCER method. **(C)** The three-dimensional stereoscopic image of the fusion result shows the STN nucleus and its subregions, electrode positions, and contact locations. The VTA is calculated based on the stimulation parameters and visualized (red part). **(D,E)**: Lead-Group analysis demonstrates the positions of the electrodes and corresponding active contacts (red markers) within the STN from anterior **(D)** and superior **(E)** views. Orange represents the sensorimotor region, blue represents the associative region, and yellow represents the limbic region. The 7-T *ex vivo* 100-mm brain atlas was used to generate the image backdrop.

The Lead-DBS toolbox (V.2.6)^1^ was used to reconstruct electrode locations for each patient (Horn et al., 2019). First, patients’ postoperative thin-slice CT scans were co-registered and fused with preoperative MRI using the Advanced Normalization Toolbox (ANTs). Images were non-linearly normalized to the Montreal Neurological Institute (MNI) space (MNI ICBM 2009b NLIN, Asym) (Avants et al., 2008) after correcting for brain shift using the Coarse mask (Schönecker et al., 2009). DBS electrode leads were reconstructed postoperatively using the PaCER algorithm (Husch et al., 2018), and deviations were manually optimized. The electrodes were then visualized in the DISTAL atlas (DISTAL minimal atlas, Ewert et al., 2018). All patients received monopolar stimulation, and the three-dimensional coordinates of the active contacts in MNI space were extracted from the reconstructed electrode models. The volume of tissue activated (VTA) was estimated in each patient’s native space using the finite element method. Based on the stimulation parameters, the VTA was calculated using the SimBio/fieldTrip method (Vorwerk et al., 2018) with the Iso2Mesh toolbox, enabling VTA visualization (Horn et al., 2017b). To generate binary VTAs, the gradient vector magnitude distribution was thresholded at 0.2 V/mm. Furthermore, the Lead-Group module was used to reconstruct patients’ 3D electrode positions in MNI standard space postoperatively. The VTA generated by the electrodes within the STN was calculated based on the active contacts and stimulation parameters. The overlap between the VTA and the STN, as well as the overlap of the VTA with the motor, associative, and limbic regions of the STN, were quantified (see Figure 1).

In this study, sweet spot analysis was performed at the voxel level. An N-image was created by counting the number of intersecting VTAs in each voxel, with voxels covered by less than 20% of VTAs excluded to ensure data validity and reliability. Each voxel within the relevant VTA was assigned a change in clinical scale scores (e.g., cognitive and emotional scores) to generate a mean effect map, reflecting changes in the patient’s cognitive and emotional function. The mean change score for each voxel was then calculated to visualize the stimulation effects across different brain regions. Voxel-wise statistical testing was applied to generate a *p*-value map assessing the significance of the effects. A two-sided Wilcoxon signed-rank test was used to detect significant (*P* < 0.05) differences in mean change scores from zero. Positive voxels (sweet) were defined as those with a mean change score significantly greater than zero, indicating beneficial effects, while negative voxels (sour) were defined as those with a mean change score less than zero, suggesting potential deterioration. VTAs with larger overlap with the sweet spots and less overlap with the sour spots would be associated with better clinical improvement. The *p*-values for each voxel were adjusted using false discovery rate (FDR) correction for multiple comparisons at an α-level of 0.05, with only those voxels surviving this statistical threshold retained for subsequent analysis. Leave-one-out cross-validation (LOOCV) was applied to validate this model.

To explore the relationship between stimulation sites and clinical outcomes, we performed fiber tracking and structural connectivity analysis using a Parkinson’s disease (PD) connectome derived from the Parkinson’s Progression Markers Initiative (The Parkinson Progression Marker Initiative [PPMI], 2011).^2^ This normative connectome, widely utilized in DBS research, enabled the estimation of structural connections between each patient’s VTA and brain regions. White matter tracts intersecting VTAs were identified using the Lead-DBS fiber-filtering method. Whole-brain tractography was conducted in DSI Studio with a generalized q-sampling approach, generating 20,000 fibers per subject. Fiber density maps were created by counting the number of fibers passing through each voxel, and only fibers present in at least 20% of patients’ VTAs were included for analysis. A “Fiber T-score” was calculated for each fiber tract by performing two-sample *t*-tests to compare clinical outcomes (changes in HAMA, HAMD, and MoCA scores) between VTAs connected to specific tracts and those that were not. Positive T-scores indicated improved outcomes linked to those tracts, while negative T-scores were associated with poorer outcomes. The top 30% of fibers by absolute T-score were visualized to highlight key pathways. To assess the predictive accuracy of the structural connectivity findings, leave-one-patient-out cross-validation (LOOCV) and k-fold cross-validation (*k* = 10) were applied.

### 2.5 Statistical analysis

Statistical analysis and descriptive analysis were performed using SPSS 27.0 (IBM Corp.), and a correlation scatter plot was generated using GraphPad Prism 9. The Shapiro–Wilk test was employed to assess the normality of the data. Continuous variables were expressed as mean ± standard deviation (x̄ ± SD) or median (1st Quartile, 3rd Quartile) [M (Q1, Q3)]. Depending on the normality of the data distribution, paired *t*-tests or Wilcoxon signed-rank tests were conducted on self-before-after control data to identify significant changes compared to baseline. Spearman’s rank correlation analysis was used to explore the relationship between improvement rates in anxiety and depression scores and the coordinates of active contacts, as well as to investigate the relationship between the improvement rate of clinical scale scores and the overlap of VTA within the STN and its subregions. All statistical tests were two-tailed, and a *P*-value < 0.05 was considered statistically significant.

## 3 Results

### 3.1 Patient characteristics, clinical outcomes, and programming parameters

This study included 56 patients (34 males and 22 females), with detailed demographic characteristics summarized in Table 1. At 6 months post-surgery, the UPDRS-III, PDQ-39, HAMA, and HAMD scores were significantly lower compared to the preoperative baseline (*p* < 0.05). Additionally, the levodopa equivalent daily dose (LEDD) decreased from 725.85 (527.50, 972.06) mg at baseline to 225.00 (200, 300) mg, representing a 69.00% reduction (*p* < 0.001). During the 6-month follow-up, all patients received monopolar stimulation. The average stimulation parameters for both hemispheres were as follows: voltage, 2.50 ± 0.31 V; frequency, 128.62 ± 18.74 Hz; and pulse width, 64.29 ± 6.64 μs. Detailed data are presented in Table 1.

**TABLE 1:** Summary of the patient baseline characteristics, clinical outcomes, and programming parameters at 6 months post-surgery.

Variable	Baseline	6-month post DBS	Z/*t*	*p*
Sex (male/female)	34/22	–	–	–
Age at surgery (year)	61.64 ± 8.57	–	–	–
Age at PD onset (year)	51.59 ± 8.77	–	–	–
Disease duration (year)	9.97 ± 5.45	–	–	–
Hoehn–Yahr stage	3.0 (3.0, 4.0)	–	–	–
UPDRS-III	44.00 (32.25, 54.75)	25.50 (19.50, 33.50)	−6.512	< **0.001**
LEDD	725.85(527.50, 972.06)	225.00 (200, 300)	−6.431	< **0.001**
PDQ39	50.27 ± 19.30	27.73 ± 14.65	7.473	< **0.001**
HAMA	7.50 (5.00, 11.00)	5.50 (2.00, 8.00)	−4.019	< **0.001**
HAMD	10.00 (6.00, 11.75)	6.00 (3.00, 9.50)	−3.171	**0.002**
DBS amplitude (V)	–	2.50 ± 0.31	–	–
DBS frequency (Hz)	–	128.62 ± 18.74	–	–
DBS pulse width (μs)	–	64.29 ± 6.64	–	–

UPDRS-III, Unified Parkinson’s Disease Rating Scale Part III; LEDD, levodopa equivalent daily dose; PDQ39, 39-item Parkinson’s Disease Questionnaire; HAMA, Hamilton Anxiety Scale; HAMD, Hamilton Depression Scale. Boldface type indicates statistical significance.

### 3.2 Impact of active contact location on anxiety and depression outcomes in PD patients

**FIGURE 2:**
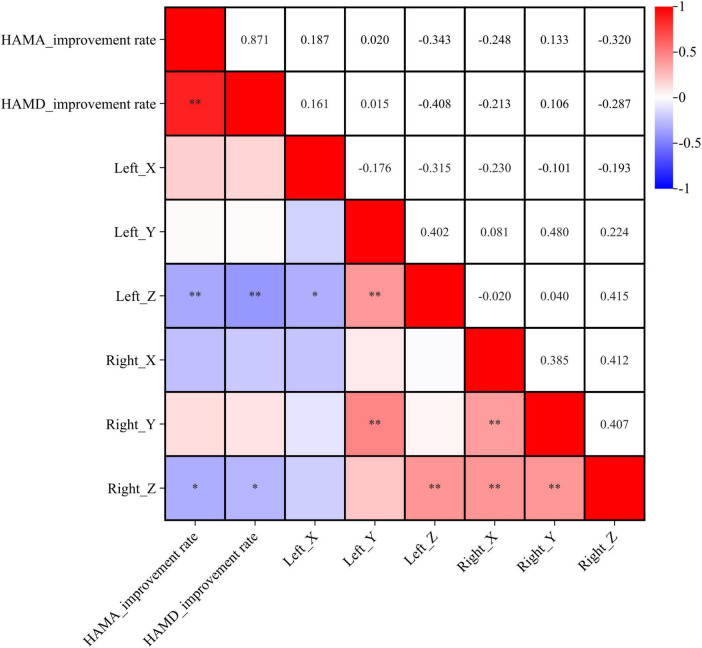
Correlation analysis between coordinates location and symptom improvement rate. X, Y, and Z represent the X, Y, and Z axes in MNI standard space. *p < 0.05, **p < 0.01.

We analyzed the correlation between coordinate positions and improvement rates in anxiety and depression. The improvement rate in HAMA scores was negatively correlated with the Z-axis coordinates of the active contacts (left: *r* = −0.343, *p* < 0.01; right: *r* = −0.320, *p* < 0.01). The improvement rate in HAMD scores was also negatively correlated with the Z-axis coordinates of the active contacts (left: *r* = −0.408, *p* < 0.05; right: *r* = −0.287, *p* < 0.05). These findings suggest that the improvement in anxiety and depressive symptoms after STN DBS in PD patients is associated with more ventral locations of the active contacts (see Figure 2).

### 3.3 Sweet spot analysis

**FIGURE 3:**
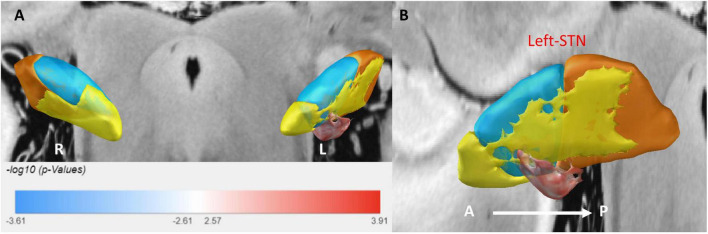
3D illustration of the sweet spot, with red voxels indicating areas correlated with improved HAMA scores. **(A)** Anterior view of sweet spot analysis showing voxels distributed exclusively in the left STN. **(B)** Lateral view of the sweet spot in the left STN. A, anterior; P, posterior. Deeper colors signify stronger correlations.

**FIGURE 4:**
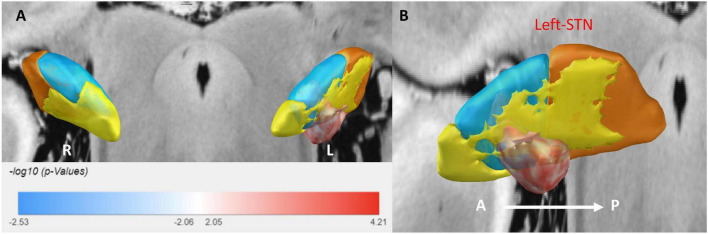
A 3D illustration of the sweet spot, with red voxels indicating areas correlated with improved HAMD scores. **(A)** Anterior view of sweet spot analysis showing voxels distributed exclusively in the left STN. **(B)** Lateral view of the sweet spot in the left STN. A, anterior; P, posterior. Deeper colors signify stronger correlations.

Sweet spot mapping was performed using Lead-DBS to assess the relationship between individual VTAs and changes in HAMA and HAMD scores at the group level. The “sweet spot” was defined as the stimulation region associated with clinical symptom improvement, while the “sour spot” referred to the region linked to symptom worsening. Positive voxels (sweet spots) were visualized in red, whereas negative voxels (sour spots) were depicted in blue. Red indicated a positive correlation, while blue represented a negative correlation. The intensity of the color reflected the strength of statistical significance. Only the sweet spot in the left subthalamic nucleus (STN) passed the false discovery rate (FDR) test (see Figures 3, 4).

For anxiety (HAMA), the sweet spot localized to the ventrocentral portion of the left STN. This spatial pattern was robustly validated via leave-one-out cross-validation (LOOCV; *R* = 0.40, *P* = 0.001). Similarly, for depression (HAMD), the sweet spot localized to the ventrocentral portion of the left STN, confirmed by LOOCV (*R* = 0.28, *P* = 0.019) (see Supplementary Figure 1).

### 3.4 Relationship between VTA and clinical improvement rates

**FIGURE 5:**
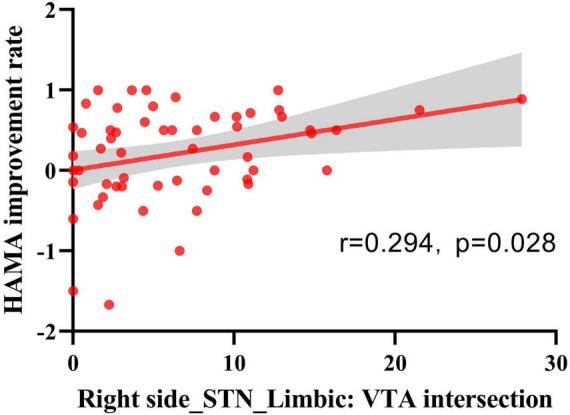
Spearman’s rank correlation analysis of VTA and HAMA score improvement rate. VTA intersections with STN-limbic regions in the right hemisphere showed a positive correlation with the HAMA improvement rate (*n* = 56, *r* = 0.294, *p* = 0.028).

We investigated the relationship between the degree of improvement in anxiety and depression at 6 months post-surgery and the VTA generated by the active contacts within the STN and its subregions. The results showed that the HAMA score improvement rate was positively correlated with the VTA in the right STN limbic region (*r* = 0.294, *p* = 0.028) (see Figure 5).

### 3.5 Structural connectivity analysis

**FIGURE 6:**
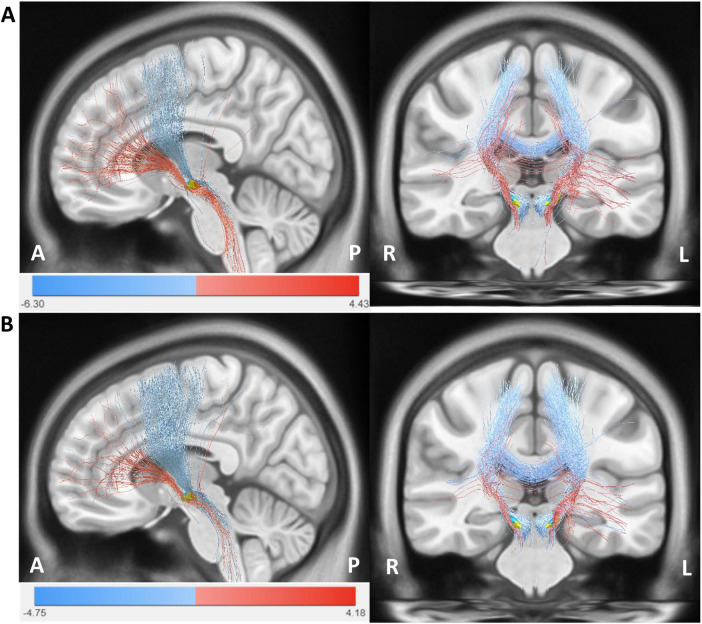
The structural connectivity network model. **(A)** Structural fiber connectome associated with anxiety (HAMA). **(B)** Structural fiber connectome associated with depression (HAMD). Red fiber bundles represent regions linked to clinical improvement, indicated by positive fiber T-scores, while blue fiber bundles denote regions associated with symptom worsening, indicated by negative fiber T-scores. The images are labeled with anterior-posterior (A-P) and right-left (R-L) orientations.

In an exploratory analysis, we plotted our results onto a normative PD connectome to illustrate potential group-level differences in structural connectivity between the VTA and cortical regions. Figure 6 shows the structural connectivity analysis seeded from bilateral VTAs across all patients. HAMA changes were positively correlated with fiber tracts connecting the VTAs in the STN to the prefrontal cortex (PFC) and negatively correlated with tracts to the sensorimotor cortex (SMC). Similarly, HAMD changes were positively correlated with fiber tracts to the PFC and negatively with tracts to the SMC. The fiber T-score–based structural connectivity model provided significant predictions in both k-fold = 10 (HAMA: *R* = 0.51, *p* < 1e-16; HAMD: *R* = 0.43, *p* < 1e-16) and leave-one-patient-out (LOOCV) (HAMA: *R* = 0.48, *p* < 1e-16; HAMD: *R* = 0.36, *p* = 0.001) cross-validation analyses. These results indicate that connectivity to the PFC is associated with symptom improvement, while connectivity to the SMC is linked with symptom deterioration (see Supplementary Figure 2).

## 4 Discussion

It is widely acknowledged that STN DBS can improve motor dysfunction, effectively reduce the dosage of dopaminergic medications, and enhance the quality of life in PD patients (Hariz and Blomstedt, 2022, Hacker et al., 2020, Castrioto et al., 2022). Consistent with previous studies, our patients demonstrated significant improvements in motor function, quality of life, and daily medication requirements after STN DBS. Non-motor symptoms in PD patients, including emotional symptoms, can negatively impact their daily life and mental health and are receiving increasing attention. While numerous studies have investigated the optimal stimulation sites for improving motor symptoms through DBS (Tödt et al., 2022, Horn et al., 2017a), limited research has been conducted on sweet spot analysis and structural connectivity analysis for emotional outcomes in patients. This study prospectively followed 56 PD patients who underwent bilateral STN-DBS for six months, systematically evaluating its therapeutic effects on emotional symptoms. Individual electrode positions and volumes of tissue activated (VTA) were reconstructed, and group-level analyses were conducted to identify optimal stimulation sites. Based on these findings, structural connectivity analyses were further performed, and the results were subsequently correlated with changes in emotional symptoms.

Emotional disorders, particularly anxiety and depression, are highly prevalent in PD patients. A meta-analysis showed that PD patients experienced mild to moderate improvements in anxiety and depression severity and an overall reduction in negative emotions after STN-DBS treatment (Bucur and Papagno, 2023, Cartmill et al., 2021). Our results demonstrated improvements in anxiety and depressive symptoms 6 months after DBS treatment, with improvement rates of 26.67 and 40.00%, respectively. Regarding cognition, previous studies have reported both improvements and declines in cognitive function after STN-DBS, with cognitive deterioration particularly involving declines in verbal fluency, memory, and executive function (Bucur and Papagno, 2023, Pillon et al., 2000, Mollion et al., 2011, Parsons et al., 2006, Wu et al., 2014, Merkl et al., 2017). However, some studies have found no significant differences in overall cognitive scores in PD patients 2 years after STN DBS compared to preoperative scores (Georgiev et al., 2021, Hong et al., 2023). Our results revealed a slight improvement in overall cognitive function 6 months after STN DBS compared to the preoperative state. This suggests that DBS may have a positive impact on cognitive function, possibly due to a combination of factors such as the modulation of motor symptoms by STN DBS, its influence on cognitive-related neural circuits, and the alleviation of anxiety and depressive symptoms.

Neuropsychiatric symptoms in patients after STN-DBS largely depend on the location of effective contacts within the STN and parameter settings. The location of active contacts and stimulation parameters vary among patients, and their clinical improvements also differ considerably. Dafsari and colleagues’ study (Dafsari et al., 2018) indicated that stimulation in the medial, anterior, and ventral regions of the STN was significantly associated with more beneficial non-motor symptoms, including anxiety and depression. Petry-Schmelzer et al. (2019) showed that improvements in mood/apathy were related to neurostimulation in the ventromedial limbic and sensorimotor subregions of the STN. Zhang et al. (2022) found that the closer the electrode stimulation was to the ventromedial limbic region of the STN, the more significant the improvement in anxiety and depressive symptoms in PD patients. Liang et al. (2023) demonstrated that stimulation in the ventral STN was more favorable for improving anxiety symptoms in PD patients. Similar to many previous studies, our research demonstrated that deep brain stimulation (DBS) targeting the more ventral regions of the subthalamic nucleus (STN) was significantly associated with greater improvements in anxiety and depressive symptoms. Specifically, improvements in anxiety showed a positive correlation with the VTA in the limbic region of the right STN. Our sweet spot analysis had the FDR correction applied to its results. Following correction, only the voxels in the ventrocentral region of the left STN were found to retain statistical significance and were significantly associated with emotional improvement. This indicates that, although a general trend of emotional improvement might be present across the ventral STN, the most robust and statistically reliable effects were concentrated in the ventrocentral region of the left STN. This outcome may be attributed to the relatively small sample size of the dataset or to a lateralized effect of DBS in processing emotions (Lin et al., 2021). This spatial pattern was robustly validated through leave-one-out cross-validation (LOOCV), confirming the reliability of ventral STN stimulation in modulating mood symptoms. These findings support the tripartite functional concept of the STN, with the limbic subregion located in the medial, anterior, and ventral aspects of the STN, connected to brain regions involved in emotional regulation (such as the amygdala, nucleus accumbens, ventral striatum, mediodorsal thalamic nucleus, and paralimbic and limbic cortices) (Krack et al., 2010). Stimulation in this region is likely to enhance limbic circuit modulation, contributing to mood stabilization.

In this study, we used a Parkinson’s disease normative connectome to perform structural connectivity analysis, revealing distinct fiber pathways connecting the STN-DBS-activated tissue volume (VTA) to various cortical regions, particularly to the prefrontal cortex (PFC) and the sensorimotor cortex (SMC). The PFC is the anterior region of the frontal lobe, known for its critical role in both cognitive functions and emotional regulation. The PFC includes subregions such as the dorsolateral prefrontal cortex (DLPFC), ventrolateral prefrontal cortex (VLPFC), orbitofrontal cortex (OFC), and anterior cingulate cortex (ACC), all of which play significant roles in emotional regulation (Kohn et al., 2014, Chen et al., 2022, Xu et al., 2019). Studies have shown that transcranial direct current stimulation (tDCS) of the PFC can reduce anxiety by modulating the response of the amygdala to threat stimuli in individuals with trait anxiety (Ironside et al., 2019), and can significantly improve depressive symptoms in patients (Woodham et al., 2025). The fiber connectivity between the subthalamic nucleus deep brain stimulation (STN-DBS) and the prefrontal cortex (PFC) exerts a significant influence on anxiety and depressive symptoms in PD patients, mediated through both structural and functional pathways. Anatomically, the hyper direct pathway links the PFC to the STN, with topographically organized projections: limbic-associated cortical regions (e.g., orbitofrontal cortex, ventromedial prefrontal cortex) project to the medial and rostral STN, while motor-related areas innervate the lateral and caudal STN (Haynes and Haber, 2013). Stimulation of the dorsolateral (motor) STN improves motor symptoms but may insufficiently address non-motor symptoms, whereas targeting the medial/ventral (limbic) STN demonstrates superior efficacy in alleviating anxiety and depression, likely due to modulation of OFC-amygdala-hippocampal circuits and normalization of dysregulated functional connectivity (FC) within fear networks (Chang et al., 2023, Le Jeune et al., 2008).

The structural connectivity analysis in this study revealed that the changes in HAMA and HAMD scores following STN-DBS were positively correlated with fiber tracts projecting to the prefrontal cortex (PFC) and negatively correlated with those projecting to the sensorimotor cortex (SMC). Specifically, fibers associated with anxiety and depression improvements traversed the ventral STN and connected in the PFC, while those associated with deterioration traversed the dorsal STN and connected in the SMC. We were able to cross-predict the outcome in a large sample of patients by using k-fold and leave-one-patient-out strategies. Functional imaging reveals that STN-DBS reduces β-band hyperconnectivity in prefrontal, sensorimotor, and limbic cortices, acutely ameliorating PD symptoms, including emotional disturbances (Conti et al., 2022). Moreover, stimulation of the right dorsolateral PFC (dlPFC) predicts depressive symptom improvement, possibly by restoring lateralized PFC activity balance, though the mechanistic underpinnings of this lateralization remain debated (Irmen et al., 2020). Long-term improvements in anxiety and depression are also linked to STN-DBS-induced metabolic changes in the OFC and enhanced GABAergic/glutamatergic neurotransmission within PFC circuits, which counteract default mode network hyperconnectivity associated with negative affect (Hare and Duman, 2020). However, stimulation may paradoxically worsen depressive symptoms if anterior-left STN regions are overactivated, highlighting the delicate balance between motor and non-motor network modulation (Irmen et al., 2020).

Our analysis revealed that stronger connectivity between the dorsal STN stimulation sites and the sensorimotor cortex (SMC) was associated with poorer emotional improvement. This negative correlation suggests that when the VTA (Volume of Tissue Activated) mainly activates the sensorimotor pathway, the emotional regulation circuit may be disrupted due to the competition between motor network activity and limbic processing. This result is consistent with the findings of Petry-Schmelzer et al. (2019), who reported that emotional improvement is poor when stimulation extends to motor-related regions.

This study had several limitations. To begin with, it is a retrospective analysis, and the electrode settings were based on clinically decided parameters rather than randomized or systematically tested protocols. Secondly, larger sample sizes and prospective studies are needed to validate these findings. Thirdly, while the use of a normative structural connectome, derived from the PPMI dataset, enabled us to explore group-level patterns of connectivity associated with DBS outcomes, we recognize its limitations in capturing the heterogeneous alterations in structural connectivity characteristic of PD (Wang et al., 2025). This approach assumes a standardized connectivity profile that may not reflect the individual variability introduced by the disease’s neurodegenerative effects. Consequently, the structural connectivity findings should be interpreted with caution and considered exploratory rather than definitive representations of patient-specific DBS effects. Additionally, Although our sweet spot and structural connectivity models demonstrated statistical significance, their R-squared values indicate that they explain only a fraction of the variance in neuropsychiatric outcomes. This underscores that while electrode placement plays a key role, additional factors like baseline neuropsychiatric status, individualized brain connectivity, and other clinical characteristics likely influence treatment response.

## 5 Conclusion

Our findings demonstrate that STN DBS can significantly improve motor symptoms and quality of life in PD patients while also having positive effects on anxiety and depression. Additionally, by mapping the proximity of fibers linking the electrode to the prefrontal cortex (PFC) and sensorimotor cortex (SMC), customized STN-DBS could predict neuropsychiatric changes. Future research should further employ brain connectomics and isolated fiber tracts to optimize the placement of DBS electrodes and utilize directional electrodes to more precisely target specific STN subregions, thereby enhancing symptom relief. The application of directional electrodes may enable DBS stimulation to not only improve motor symptoms but also focus more effectively on mood regulation, further advancing the implementation of personalized neuromodulation therapies.

## Data Availability

The original contributions presented in this study are included in this article/Supplementary material, further inquiries can be directed to the corresponding author.

## Footnotes

1
http://www.lead-dbs.org

2
www.ppmi-info.org
