# Supplementary material for: Sweet spot mapping and structural connectivity in subthalamic stimulation: predicting neuropsychiatric outcomes in Parkinson’s disease
Source: Front Neurosci. 2025 Apr 28;19:1577588. doi: 10.3389/fnins.2025.1577588 (PMC12066317; doi:10.3389/fnins.2025.1577588)
Supplement: Supplementary file 1 [file Data_Sheet_1.docx]

Supplemental material


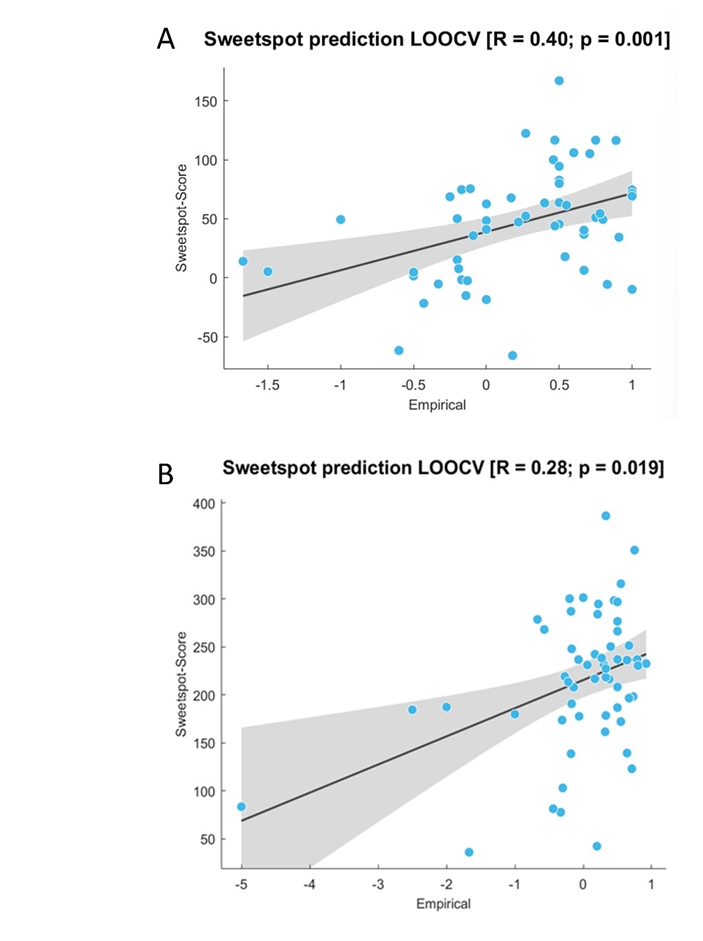


**Figure 1.** Results of sweet spot analysis were validated in a LOOCV design. Panel A illustrates the LOOCV validation for the HAMA (Hamilton Anxiety Rating Scale) sweet spot, while Panel B represents the LOOCV validation for the HAMD sweet spot.


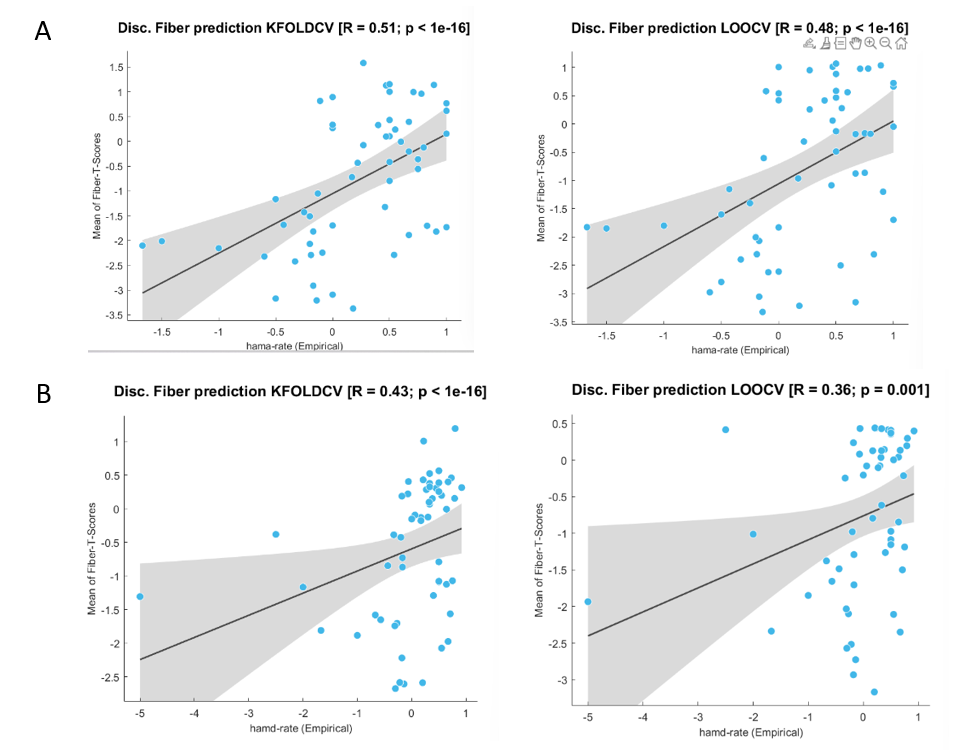


**Figure 2.** Results from predictive structural connectivity network model were validated in k-fold=10 and LOOCV design. Panels A and B show the results of the predictive structural connectivity network model validated using two cross-validation methods: k-fold (k=10) and Leave-One-Out Cross-Validation (LOOCV). Panel A displays the validation for the change in HAMA scores, while Panel B presents the validation for the HAMD scores.
